# Sensory trick effect in craniofacial dystonia as one of the possible impacts of wearing face masks during the COVID-19 pandemic

**DOI:** 10.1186/s42466-021-00123-2

**Published:** 2021-06-01

**Authors:** Frank Erbguth, Rüdiger Lange

**Affiliations:** Department of Neurology, Paracelsus Medical University – Nuremberg General Hospital, Breslauer Str. 201, 90471 Nürnberg, Germany

**Keywords:** Face mask, Craniofacial dystonia, Blepharospasm, Oromandibular dystonia, Hemifacial spasm, COVID-19, Sensory trick

## Abstract

**Background:**

The report of a patient with blepharospasm during the COVID-19 pandemic suggested a potential ameliorating effect of wearing a face mask.

**Objective:**

We prospectively evaluated a possible symptom change through wearing a face mask in all consecutive patients with craniofacial hyperkinesias in our botulinum toxin outpatient treatment cohort.

**Methods:**

Patients with craniofacial hyperkinesia were asked to rate changes of symptoms between − 2 (markedly worsened), − 1 (slightly worsened), 0 (no change), + 1 (slightly improved) and + 2 (markedly improved).

**Results:**

Of 101 patients (19 with blepharospasm [BSP], 54 with cervical dystonia [CD], 6 with oromandibular dystonia [OMD], and 22 with hemifacial spasm [HFS]) 81 (80%) rated no symptom change, 11 (11%) symptom improvement, and 9 (9%) symptom worsening. Improvements in 9 of the 82 dystonia patients (BSP, CD, OMD) consisted of a perceived decrease in dystonic activity. 33% of dystonia patients had previously noticed or used a sensory trick. Its presence turned out to be a significant predictor of improvement during mask wearing. Deteriorations were attributed from all patients to disturbing effects of the mask interacting with facial muscle overactivity. Improvements in HSF patients were attributed to the symptom-hiding nature of the mask and not to an effect on the spasm activity itself.

**Conclusions:**

Wearing a face mask did not affect self-perceived symptoms in 80% of patients with craniofacial hyperkinesis. 11% of patients reported an improvement, which occurred as sensory trick in dystonia patients and as a concealment of a stigmatizing facial expression in patients with HSF.

During April 2020, the wearing of face coverings for protection against COVID-19 infection became widespread in public spaces in Germany. As of 27th April 2020, a general mask requirement was imposed in Germany in shops and on public transport or at the workplace, and it remains in force to this day.

During a botulinum toxin treatment session on 23rd April 2020, a patient with blepharospasm (BSP) spontaneously reported, without being asked, that while he was wearing a mask, the activity of his involuntary eye closure had improved significantly. Therefore, we started to evaluate this phenomenon systematically in a prospective study during two injection cycles in all consecutive patients in our botulinum toxin treatment cohort with craniofacial movement disorders.

## Methods

All consecutive patients with different forms of chronic hyperkinetic craniofacial movement disorders under long-term botulinum toxin treatment who presented for botulinum toxin reinjections in our movement disorder outpatient clinic between 30th April 2020 und 6th August 2020 (cycle 1) were prospectively included in a structured evaluation by interview and clinical examination about possible effects of wearing a mask on the symptoms. The various craniofacial movement disorders were categorized as (1) blepharospasm (BSP), including mild Meige-Syndrome components, (2) cervical dystonia (CD), (3) oromandibular dystonia or complex lower facial dystonia (OMD), and (4) hemifacial spasm (HFS). Patients with hemifacial spams were included to serve as non-dystonic controls to get an idea of the specific vs. unspecific nature of the possible perceptions of dystonia patients.

The evaluation was repeated identically through the following injection cycle between 30th July 2020 and 20th November 2020 (cycle 2). Only patients who could be interviewed in both injection cycles were included in the evaluation.

The intensity/frequency of mask wearing was divided into four categories:

- **0 = never/very rarely:** up to a total of < 3 h per week.
- **1 = rarely:** 3–7 times per week and/or > 3 h per week
- **2 = moderate**: 1–2 times per day or > 0.5–3 h per day
- **3 = frequently**: daily and > 3 h per day.

All patients were asked: “*Did you notice any change in your symptoms caused by your [BSP, HFS, CD, OMD] while wearing the mask - or not?”. Please indicate on the scale:****− 2*** *(markedly worsened) /* ***− 1*** *(slightly worsened) /* ***0*** *(unchanged) /* ***+ 1*** *(slightly improved) /* ***+ 2*** *(markedly improved).*

In case of deterioration or improvement, the patients were asked to give a short description of what this change consisted of. The presence of a pre-existing sensory trick manoeuvre was identified from the case records and queried again during the session.

## Results

One hundred twenty patients (mean age 72.8 ± 11.6 years) could be evaluated in each of the two cycles. Because only wearing-frequency categories 1 to 3 were evaluated to exclude infrequent or non-practiced mask wearing (*n* = 19), 101 patients were included in the evaluation: 22 patients with BSP, 19 patients with HFS, 54 patients with CD, and 6 patients with OMD (Table 1). The inter-injection interval averaged 12 weeks (11–14 weeks) in both cycles.

**Table 1:** Baseline characteristics, mask wearing frequencies, and symptom changes of the evaluated patients during cycle 1

Diagnosis	n	Age y (mean, SD)	Frequency of wearing the mask (category) (n)				Frequency category 1–3 (n)	Symptom Change category (n) (−2 to + 2) and number of patients with preexisting sensory trick [n]				
			0	1	2	3		-2	-1	0	+ 1	+ 2
BSP	26	71,9 ± 8,9	4	12	6	4	**22**	1 [0]	1 [1]	17 [4]	2 [1]	1 [1]
CD	62	64,5 ± 12	8	20	18	16	**54**	1 [0]	2 [1]	48 [13]	2 [2]	1 [1]
OMD	7	70,3 ± 5,1	1	2	3	1	**6**	0	1 [0]	2 [1]	2 [1]	1 [1]
HFS	25	70,4 ± 10,6	6	10	5	4	**19**	1 [0]	2 [0]	14 [0]	1 [0]	1 [0]
	**120**		**19**	**44**	**32**	**25**	**101**	**3 [0]**	**6 [2]**	**81 [18]**	**7 [4]**	**4 [3]**

### Cycle 1

Of the 101 patients analyzed, 81 (80%) reported no changes, 9 (9%) reported deterioration, and 11 (11%) reported improvements.

27% (27 of 101) of the patients had previously used sensory tricks. None of the HFS patients had a trick, so that of the remaining 82 dystonia patients, 27 (33%) reported a trick: 7 of the 22 BSP patients (32%), 17 of the 54 CD patients (31%) and 3 of the 6 OMD patients (50%). Twenty of the Seventy-three dystonia patients (27%) with no change or deterioration used a sensory trick, while 7 of the 11 dystonia patients with improvement (64%) reported a trick, indicating a significantly higher prevalence of pre-existing sensory tricks in patients with symptom amelioration (7/11 vs 20/73).

#### Descriptions of the effects of the mask by the patients

- **Improvements in BSP, CD, and OMD (*n*** **= 9):** Dystonic activity decreases immediately or with a delay after the mask is put on.
- **Deteriorations in BSP, CD, and OMD (*n*** **= 6):** In BSP and OMD the periorbital and perioral dystonic movements are transmitted to the mask and thus amplified. In CD wearing the mask is unpleasant along with involuntary head movements and the movements of the head cause the mask to lose its correct position.
- **Improvements in HFS (*n*** **= 2):** The twitching is hidden under the mask and no longer visible, which reduces the stigmatizing effect of the disorder and thus leads to a generally greater feeling of well-being.
- **Deteriorations in HFS (*n*** **= 3):** The twitching of the facial muscles is more unpleasant because it is transmitted to the mask and thus amplified.

**Fig. 1:**
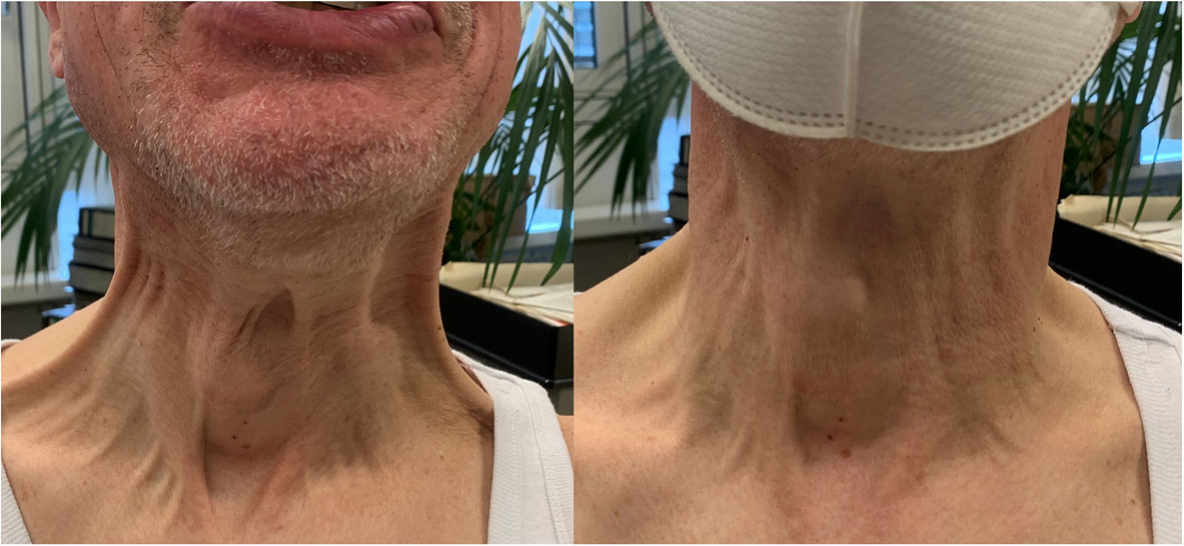
Patient with permanent complex orofacial dystonia with severe platysmal involvement before (left) and promptly after mask application: platysmal activity decreases markedly (right). A sensory trick existed in the patient: without the mask he was able to similarly ameliorate the dystonia by touching his laryngeal region

In 4 of the 9 dystonia patients with improvement, the reported change could be observed during the clinical examination (Fig. 1). The remaining 5 patients stated that the improvement occurred only after prolonged mask wearing of several minutes.

Even it was not asked explicitely, all 11 patients with improvement and all 9 patients with deteriorations reported, that the perceived effect lasted for the entire time they used the mask. The duration of use varied considerably, ranging from a few minutes during a short shopping trip to several hours during work.

### Cycle 2

Of the 101 patients evaluated in both cycles, 97 provided the same information as in the first cycle. Four patients showed a change between the examinations of the first and second cycles. One patient with HSF who had reported worsening in the first cycle stated in the second cycle that the mask no longer bothered him and no symptom worsening occurred. One patient with BSF who had reported improvement in the first cycle stated in the second cycle that there was no longer any improvement related to the mask. The improvement due to the mask during cycle 1 increasingly and permanently disappeared within 4–6 weeks during cycle 2. A similar course of disappearance of the improvement was reported by 2 patients with CD.

## Discussion

Inspired by the report of a patient with blepharospasm at the beginning of the first wave of the COVID-19 pandemic that the involuntary periocular dystonic activity had improved due to wearing a facial mask, we investigated a cohort of patients with craniofacial movement disorders attending our botulinum toxin outpatient clinic for the presence of such a phenomenon. According to our literature search, no studies have hitherto been published on this topic. While 80% of the 101 patients evaluated reported no change during mask use, improvements were observed in 11% and deteriorations in 9%. In contrast to HSF, in which the improvements and deteriorations were not due to an effect on spasm activity itself, the improvements in dystonia were due to a decrease in pathological dystonic muscle activity. The symptom deteriorations in dystonia patients were not based on an increase in muscle activity, but were caused by an unpleasant transmission of the stable muscle twitches to the mask. We interpreted the positive effect of the mask in the dystonia patients as a sensory afferent stimulation that ameliorated dystonic activity like a sensory trick. This assumption is supported by the finding that a pre-existing sensory trick turned out to be a statistically significant predictor of improvement in our dystonia patients through the wearing of a mask.

Sensory tricks involve various stimuli with resultant amelioration of dystonic muscle contractions [1, 2] by influencing aberrant central nervous sensorimotor processing and integration [3, 4]. There is a wide variability in the reported frequency of sensory tricks in patients with craniofacial dystonia ranging from 20 to 80% [5, 6]. Therefore, the 33% rate of sensory tricks in the dystonia patients in our cohort is within this range. A similar observation to our study was published in 1991 in a case report on 2 patients with cervical dystonia [7], in whom a close-fitting cardboard box on the head corrected the pathological dystonic rotation of the head to a normal position.

The interpretation that the observed phenomenon is a sensory trick is also supported by the observation that in the second cycle the alleviating effect had become habituated in three of the dystonia patients, which is characteristic of part of the sensory tricks [8].

A limitation of our study is that the data were based mainly on the information provided by the patients and the effect of the mask including its duration could only be confirmed by direct observation in 5 of the 11 patients. Making regular video recordings with a smartphone throughout the inter-injection interval by the patients could address this limitation in future studies.

## Conclusion

Wearing a face mask during the COVID-19 pandemic did not affect self-perceived symptoms in 80% in out cohort of 101 patients with hyperkinetic craniofacial movement disorders. Like healthy individuals, approximately 9% of patients perceived the mask as disturbing. Positive effects from wearing a mask occured in 10% of HFS patients by concealing stigmatizing symptoms and in 11% of dystonia patients by alleviating pathological muscle hyperactivity as a sensory trick.

## Data Availability

All data generated or analysed during this study are included in this published article (Table 1).
